# Pulmonary large cell neuroendocrine carcinoma exhibiting extensive pagetoid spread in the bronchial epithelium: A case report

**DOI:** 10.3892/ol.2014.2538

**Published:** 2014-09-16

**Authors:** HIROYUKI OGAWA, YUGO TANAKA, YU-ICHIRO KOMA, DAISUKE HOKKA, SHINYA TANE, SHUNSUKE TAUCHI, KAZUYA UCHINO, MASAHIRO YOSHIMURA, YOSHIMASA MANIWA

**Affiliations:** 1 Department of Thoracic Surgery, Kobe University Hospital, Chuo-ku, Kobe City, Hyogo 650-0017, Japan; 2 Department of Pathology, Hyogo Cancer Center, Akashi, Hyogo 673-8558, Japan; 3 Department of Thoracic Surgery, Hyogo Cancer Center, Akashi, Hyogo 673-8558, Japan

**Keywords:** large cell neuroendocrine carcinoma, pagetoid spread, lung cancer

## Abstract

Pulmonary large cell neuroendocrine carcinoma (LCNEC) is a rare and aggressive malignant tumor, which was proposed as a novel type of neuroendocrine tumor in 1991. Although it is categorized as a non-small cell lung carcinoma, the precise pathological condition is unknown due to its rare occurrence. The present study outlines the case of a patient presenting with an LCNEC that exhibited pagetoid spread from the region of the primary tumor to the bronchial epithelium (distance, >30 mm). The pagetoid spread was unconfirmed preoperatively, however, was identified by intraoperative rapid diagnosis. This caused us to suffer the perioperative decision of additional resection and resulted in an incomplete resection, as suture of the bronchus was not possible. Pagetoid spread, which is often apparent in the breast, presents as a rare pattern of infiltration of cancer cells when a massive carcinoma is identified beneath the intraepithelial spread. Although preoperative diagnosis of pagetoid spread is difficult due to its rarity and undefined clinical features, it is important for surgeons and pathologists treating lung cancer patients to be aware of potential pagetoid spread in the thoracic region.

## Introduction

Travis *et al* (1) proposed pulmonary large cell neuroendocrine carcinoma (LCNEC) as a novel category of neuroendocrine tumor in 1991. Although certain studies have reported cases of pulmonary LCNEC (1,2), its clinicopathological features have not been fully characterized due to its rarity. The present study describes a case of pulmonary LCNEC exhibiting extensive pagetoid spread in the bronchial epithelium. Due to the unexpected nature of the pagetoid spread, difficult surgical decisions were determined during the initial surgical procedure. Written informed consent was obtained from the patient.

## Case report

**Figure 1:**
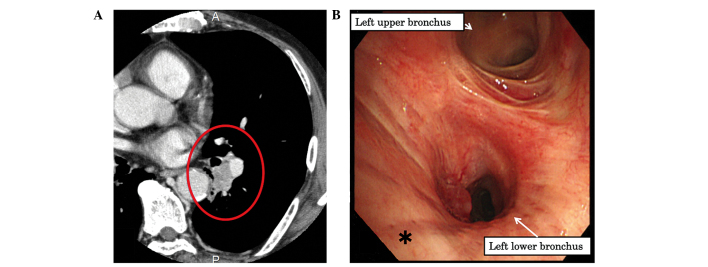
(A) Chest computed tomography revealing a 25×21-mm tumor in the hilum of the left lower lobe. (B) The tumor and the left main bronchus in the area marked by the asterisk were biopsied to estimate the tumor histology and the invasive area within a few millimeters from the tumor.

**Figure 2:**
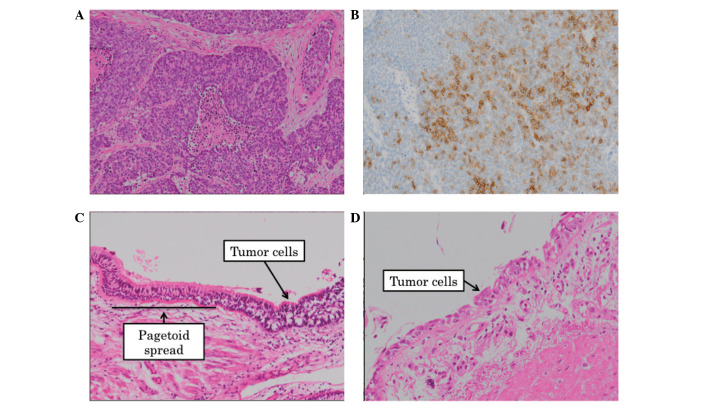
(A) Microscopically, the tumor cells exhibited neuroendocrine architectural features, such as trabecular and rosette patterns. Mitotic counts were 100 cells per 10 high-power fields (hematoxylin and eosin [H&E] stain; magnification, ×100). (B) Immunohistochemical staining demonstrated that tumor cells were positive for neural cell adhesion molecule (magnification, ×100). (C) Tumor cells demonstrated pagetoid spread in the bronchial epithelium (H&E stain; magnification, ×200). (D) Pathological examination of the biopsied specimen in the area marked by the asterisk in Fig. 1B. Preoperatively, this site was considered to be intact; however, on postoperative review it was identified that tumor invasion had previously occurred (H&E stain; magnification, ×200).

In February 2010, a 75-year-old male presented to Hyogo Cancer Center (Akashi, Japan) with an abnormal chest X-ray shadow. Chest computed tomography (CT) revealed a 25×21-mm tumor in the hilum of the left lower lobe without any indication of lymphadenopathy or metastasis (Fig. 1A). Positron emission tomography-CT demonstrated a marked accumulation of fluorodeoxyglucose in the tumor, with a maximum standardized uptake value of 7.82. This indicated that the lesion was a type of lung cancer, stage cT_1b_N_0_M_0_. Staging was designated using the TNM classification according to the 7th edition of the American Joint Committee on Cancer Staging Manual and the Revised International System for staging lung cancer (3). Spirometry determined the patient’s forced vital capacity to be 3.40 liters, which was 103.1% of the predicted value; the forced expiratory volume in 1 sec was 1.84 liters and 68.8% of the predicted value. A bronchoscopy examination demonstrated that the tumor was completely obstructing the B6 left lower lobe. The tumor and the area around the second carina closer to the carina in the bronchial airway were biopsied to estimate the nature of the invasive area (Fig. 1B). Pathology revealed a suspected LCNEC with the central side appearing to be intact (Fig. 2A and B). A left lower sleeve lobectomy with mediastinal lymph node dissection was planned.

During surgery, there were no signs of macroscopic bronchial invasion by the tumor. Based on the preoperative diagnosis, the left lower lobe, including aspects of the left main bronchus was resected to achieve a sufficient surgical margin. Although the central bronchial excision line was >25 mm away from the tumor, examination of frozen sections of the central segment revealed the presence of tumor cells. Consequently, further resection of the left main bronchus, 10 mm closer to the carina, was performed; however, microscopy revealed that tumor cells remained. A pneumonectomy was considered, however, a complete resection was not guaranteed due to uncertainty regarding the extent of the tumor spread. Considering the age and lung function of the patient, a pneumonectomy was not performed and the surgery was concluded with a sleeve lobectomy and was determined to be a microscopically incomplete resection.

Pathology of the postoperative sample revealed that the tumor was a stage pT_1b_N_0_M_0_ LCNEC, pathologic stage 1A (3) and that there was extensive one layer invasion to the central side in the bronchial epithelium, termed pagetoid spread (Fig. 2C). Careful review of the biopsied specimen during a preoperative bronchoscopy revealed that the tumor invasion was already present as pagetoid spread surrounding the second carina (Fig. 2D).

Following surgery, a bronchoscopy was performed and the bronchial tissues between the trachea and the anastomotic site were biopsied; however, no tumor cells were identified. It was presumed that the tumor cells were present only at the very near-side of the anastomotic site. Subsequently, adjuvant radiotherapy (25 fractions of 50 Gy) was administered according to the National Comprehensive Cancer Network guidelines (4). The patient remained healthy without any signs of recurrence for 30 months following the surgery, as determined by systemic work-up including enhanced chest CT, brain magnetic resonance imaging and bone scintigraphy.

## Discussion

The term pagetoid spread refers to a rare pattern of infiltration of cancer cells when a massive carcinoma is identified beneath the intraepithelial spread. Paget’s disease of the breast is well known to exhibit this pattern of infiltration, however, it is also observed in extramammary areas, such as the genital region (5). With regard to the lungs, diffuse idiopathic pulmonary neuroendocrine cell hyperplasia (DIPNECH) demonstrates a similar type of progression (6). Although DIPNECH is considered to be a precursor lesion of carcinoid tumors (6), the present case was pathologically diagnosed as a pulmonary LCNEC. Furthermore, 25 mm of macroscopically tumor-free bronchial margin is considered to be safe to perform a lung cancer resection (7). Despite the bronchial margin from the tumor edge being >35 mm, tumor cells remained in the present case. This case was exceptional due to the rare spreading pattern and the extent of the tumor invasion.

Preoperative diagnosis was complex for this rare spreading pattern due to the single layer of tumor cells in the bronchial epithelium. An accurate assessment of the tumor invasive area may contribute to a more effective therapeutic strategy. Although it is difficult to determine a final diagnosis of LCNEC using only biopsy specimens (8), a diagnosis of ‘possible LCNEC’ has been proposed by the International Association for the Study of Lung Cancer Study Group, the American Thoracic Society and the European Respiratory Society Classification for Small Biopsies (9). When biopsy specimens meet the criteria of ‘possible LCNEC’, clinicians and surgeons should pay particular attention to the bronchial margin from the tumor, as LCNEC may manifest as extensive pagetoid spread.
